# Clarification of the infection pattern of *Xanthomonas citri* subsp. *citri* on citrus fruit by artificial inoculation

**DOI:** 10.1186/s13007-024-01190-7

**Published:** 2024-05-09

**Authors:** Lian Liu, Xin Liu, Lingyi Liu, Tao Zhu, Rongchun Ye, Hao Chen, Linglei Zhou, Guang Wu, Limei Tan, Jian Han, Ronghua Li, Xianfeng Ma, Ziniu Deng

**Affiliations:** 1 National Center for Citrus Improvement-Changsha, College of Horticulture, Hunan Agricultural University, Changsha, 410128 China; 2 Engineering Research Center for Horticultural Crop Germplasm Creation and New Variety Breeding, Ministry of Education, Changsha, 410128 China; 3 Chenzhou Institute of Agricultural Science, Chenzhou, 423000 China; 4 NanLing Institute of Citrus Industry, Chenzhou, 423000 China; 5 Hunan Academy of Agricultural Sciences, Hunan Horticultural Research Institute, Changsha, 410125 China; 6 Comprehensive Experimental Station of Navel Sweet Orange in South Hunan, Chenzhou, 424200 China

**Keywords:** Citrus fruit, Citrus canker disease, Fruit development, *Xcc* infection pattern on fruit, *Xcc* artificial inoculation

## Abstract

**Background:**

Citrus canker is a significant bacterial disease caused by *Xanthomonas citri* subsp. *citri* (*Xcc*) that severely impedes the healthy development of the citrus industry. Especially when citrus fruit is infected by *Xcc*, it will reduce or even lost its commercial value. However, due to the prolonged fruiting cycle and intricate structure, much less research progress had been made in canker disease on fruit than on leaf. In fact, limited understanding has been achieved on canker development and the response to *Xcc* infection in fruit.

**Results:**

Herein, the progression of canker disease on sweet orange fruit was tracked in the field. Results indicated that typical lesions initially appear on the sepal, style residue, nectary disk, epicarp, and peduncle of young fruits after petal fall. The susceptibility of fruits to *Xcc* infection diminished as the fruit developed, with no new lesions forming at the ripening stage. The establishment of an efficient method for inoculating *Xcc* on fruit as well as the artificial inoculation throughout the fruit's developmental cycle clarified this infection pattern. Additionally, microscopic observations during the infection process revealed that *Xcc* invasion caused structural changes on the surface and cross-section of the fruit.

**Conclusions:**

An efficient system for inoculation on citrus fruit with *Xcc* was established, by which it can serve for the evaluation of citrus germplasm for canker disease resistance and systematic research on the interactions between *Xcc* and citrus fruits.

## Background

Citrus is one of the most important cash crops in the world and occupies the leading position of fruit production globally. However, the global citrus industry faces significant threats, with citrus canker being one of the most serious challenges and a key worldwide quarantine disease [1–3]. The pathogen responsible for citrus canker is a Gram-negative bacterium, *Xanthomonas citri* subsp. *citri (Xcc)*, which invades the intercellular space through natural openings like stomata and lenticels or through mechanical wounds caused by insect bites or strong wind and heavy rain [4, 5].

Young citrus tissues, including leaves, shoots, and fruits, are susceptible to *Xcc* infection and can develop typical lesions, although the canker symptoms may differ slightly due to structural differences among the them. When a large area of infection occurs, leaves fall off, branches die, and, more seriously, fruits heavily drop, resulting in reduced yield [6–9]. The surface blemishes on fruits with canker also diminish their market value, directly affecting farmers’ income. Furthermore, because the pathogenic bacteria are able to spread with the fruit, in the canker infected areas, citrus fruits cannot be sold in any market where citrus production is present and canker is not epidemic, due to quarantine laws. This seriously restricts the market development of citrus in infected areas. Therefore, prevention and control of fruit canker disease is a top priority for citrus producers.

At present, citrus fruits from widely cultivated cultivars, such as sweet oranges including navel oranges (*Citrus sinensis*), pomelos (*C. grandis*) and grapefruits (*C. paradisi*), as well as the popular hybrid ‘Orah mandarin’, are highly susceptible to citrus canker. In order to control the spread of *Xcc*, bactericides such as copper chemicals are frequently sprayed [7, 10, 11], which not only increases the production costs but also violates the citrus sustainable development. Researchers are seeking more efficient solutions to this problem [12], necessitating a clear definition of the occurrence and transmission pattern of citrus fruit canker for precise prevention and control. Studies reported that *Xcc* infection on citrus tissues was limited to certain developmental stages; when the tissues were too young, the pathogenic bacteria could not invade because the stomata were not well formed [2, 4, 5]. As tissues mature, invasion became difficult due to the increase of physical barriers like the cuticle on the surface [13–15]. In our previous studies, the leaves of at half their maximum area were found to be the most sensitive to canker disease [16]. Additionally, field observations showed that the fruit during the expansion period (90 to 120 days after fruit set) was highly susceptible to canker pathogen [4]. Climate is an important factor affecting the occurrence and prevalence of canker disease [17, 18]. Under high temperature and humidity conditions, the symptoms occur dramatically. *Xcc* can proliferate in the tissue at temperatures ranging from 6 to 30 °C, with the optimal temperature at 25 to 30 °C, and with the most favorable humidity being over 50%. If the environmental conditions are not suitable, it takes at least 60 days to present visible symptoms. Based on these knowledges, it seems that the summer shoots and the fruits in expansion are easily infected by *Xcc*, but with warmer global temperature, the spring shoots also become a node for canker disease outbreaks [19].

Compared to leaves, the structure of citrus fruit is more spatial and composted with epicarp, albedo, segment membrane, and juice sacs from the outside to the inside, forming a spherical three-dimensional structure [20–22]. Furthermore, the fruit development cycle is lengthy, encompassing three primary stages: cell division, fruit expansion, and ripening [23]. The prolonged development cycle, complex structure, and the absence of an efficient system for *Xcc* inoculation have significantly limited and impeded researches on citrus fruits related to *Xcc* infection. However, the industry primarily suffers direct economic damage from citrus canker disease affecting the fruits. Preventing and controlling damages to fruit caused by *Xcc* are crucial for the economic success in citrus industry [24]. Currently, researchers have primarily focused on studying canker disease in the leaves, with little known about the pattern of fruit infection and response to *Xcc*. In this study, we conducted in vitro and in vivo *Xcc* inoculation on fruits to develop efficient methods for evaluating disease resistance and to analyze the infection patterns and responses to *Xcc* invasion in fruits.

## Methods

### Plant materials

For inoculation in vitro, young fruits were harvested from citrus canker susceptible genotype, ‘Bingtang’ (BT) sweet orange (*C. sinensis*), and stored at 4 ℃. Fruits at 60 days after flowering (DAF) were collected for the tests of inoculation method and inoculum concentration, and those at 80 DAF were picked for the incubation temperature experiments following inoculation. Fruits with 2 to 3 cm long retained fruit stalks, of uniform size and free from surface pests and disease were chosen for the test. The fruit surfaces were wiped clean with sterile water prior to inoculation.

For inoculation in vivo, fruits at the expansion stage, at about 120 DAF, from six-year-old BT sweet orange and ‘Eureka’ lemon (*C. limon*) trees were treated.

### *Xcc* culture and pathogenicity assays

The conserved *Xcc* strain, DL509 originated from diseased leaves displaying canker symptoms in a BT sweet orange orchard in Chenzhou City, Hunan Province, was utilized in this study. The pathogen has been confirmed for its pathogenicity. The GFP-labled strain eGFP*-Xcc*, developed through triple hybridization of wild strain DL509 with DH5α containing plasmid pMP2463 and pRK2013 [25], was used to study the *Xcc* infection process in citrus fruit. The *Xcc* strains stored at -80 °C were incubated on Luria–Bertani (LB) solid medium for 2 days, and single colonies were picked and cultured in LB liquid medium at 30 °C and shaked at 220 rpm for 18 h. The strain was resuspended in sterile distilled water to an optical density (OD) of 0.6, equivalent to a bacterial suspension concentration of 10^9^ cfu/mL, and then diluted for use.

### In vitro inoculation of *Xcc* on fruit

To determine the suitable conditions for the inoculation with *Xcc* on citrus fruit, an exploratory experiment including inoculation method, inoculum concentration of and incubation temperature was set up. Four inoculum concentration gradients, ranging from 10^5^ to 10^8^ cfu/mL, were tested. Four inoculation methods were practiced with 10^8^ cfu/mL *Xcc* inoculum, i.e. inoculating fruits by pinprick, soaking, pinprick + soaking, and soaking with silwet. The specific operational methods are as follows: (1) Pinprick inoculation involved using a large-headed needle dipped in bacterial suspension and then punctured the equatorial part of the fruit in four directions, creating 6 holes per direction, with a puncture depth of approximately 1 mm. (2) Soaking inoculation was performed by submerging all the fruits in sufficient amount of *Xcc* suspension for 1 min, and then placing them in the culture container once the surface dried. (3) Pinprick + soaking inoculation just combined pinprick and soaking inoculation methods, exactly the fruit was inoculated by pinprick as described above in method (1) followed by soaking in *Xcc* suspension as mentioned in method (2). (4) Soaking with silwet inoculation was similar to the above method (2) modified by the addition of the surfactant Silwet L-77 (GE Healthcare Bio-Science AB) in the *Xcc* bacterial suspension to a final concentration of 0.02%. For incubation temperature trials, fruits inoculated with 10^8^ cfu/mL of *Xcc* were incubated under 25 ℃ and 30 ℃ separately. Ten fruits with fruit stalks were inoculated per treatment, placed in a container of water, and incubated in a light incubator at 85% RH, 12,000 lx in light intensity with 12 h of light and 12 h of darkness. Fruit abscission rate and sympom incidence were recorded.

### In vivo inoculation of *Xcc* on fruit

In vivo inoculation tests were conducted on two susceptible citrus genotypes, BT sweet orange and ‘Eureka’ lemon, using three methods including soaking, pin-prick + soaking, and soaking with silwet. Five fruits per genotype/method combination were inoculated with 10^8^ cfu/mL bacterial suspension as above described, and the trees bearing the treated fruits were kept in a pathology greenhouse at 30 to 35 ℃.

### Inoculation of *Xcc* throughout the whole fruit developmental cycle

Inoculation was performed on citrus flowers (full petal flower stage, bloom stage) ovaries, and on fruits at young, expanding and ripening stages. Ripening fruits were in vivo inoculated, while the other stages were in vitro inoculated. During all periods, inoculation was conducted by soaking with silwet using 10^8^ cfu/mL bacterial suspension. Additional pinprick and rubbing treatments were applied to the ripening fruit inoculation. In each period, 5 to 10 fruits (or flowers) were treated and incubated in a pathology greenhouse or incubator.

### Scanning electron microscope observation

To monitor fruit stomata formation, ovaries from flowers of BT sweet orange at different stages were sampled. Epicarp, style, flower disc, sepal, and fruit stalk were cut into no larger than 3 mm^2^ pieces and immediately fixed with electron microscopy fixative (Servicebio, Wuhan, China) at 4 ℃. The samples were observed and photographed with a JSM-6380LV scanning electron microscope (SEM; Jeol, Tokyo, Japan).

### Observation of *Xcc* invasion process

BT sweet orange fruits at 30 DAF were inoculated with 10^8^ cfu/mL eGFP*-Xcc* bacterial solution by soaking with silwet before observation in serial sections. The samples were sectioned using a freezing microtome (Leica CM1900, Berlin, Germany) at a thickness of 20 μM. Sections were examined using fluorescence microscopy (Zeiss Axio Imager M2, Oberkochen, Germany) immediately after sectioning.

### Data processing

Data processing was performed using Microsoft Excel (16.46) and GraphPad Prism 8 statistical analysis software.

## Results

### The earliest canker symptoms on citrus fruit observed at blossom fall stage

**Fig. 1:**
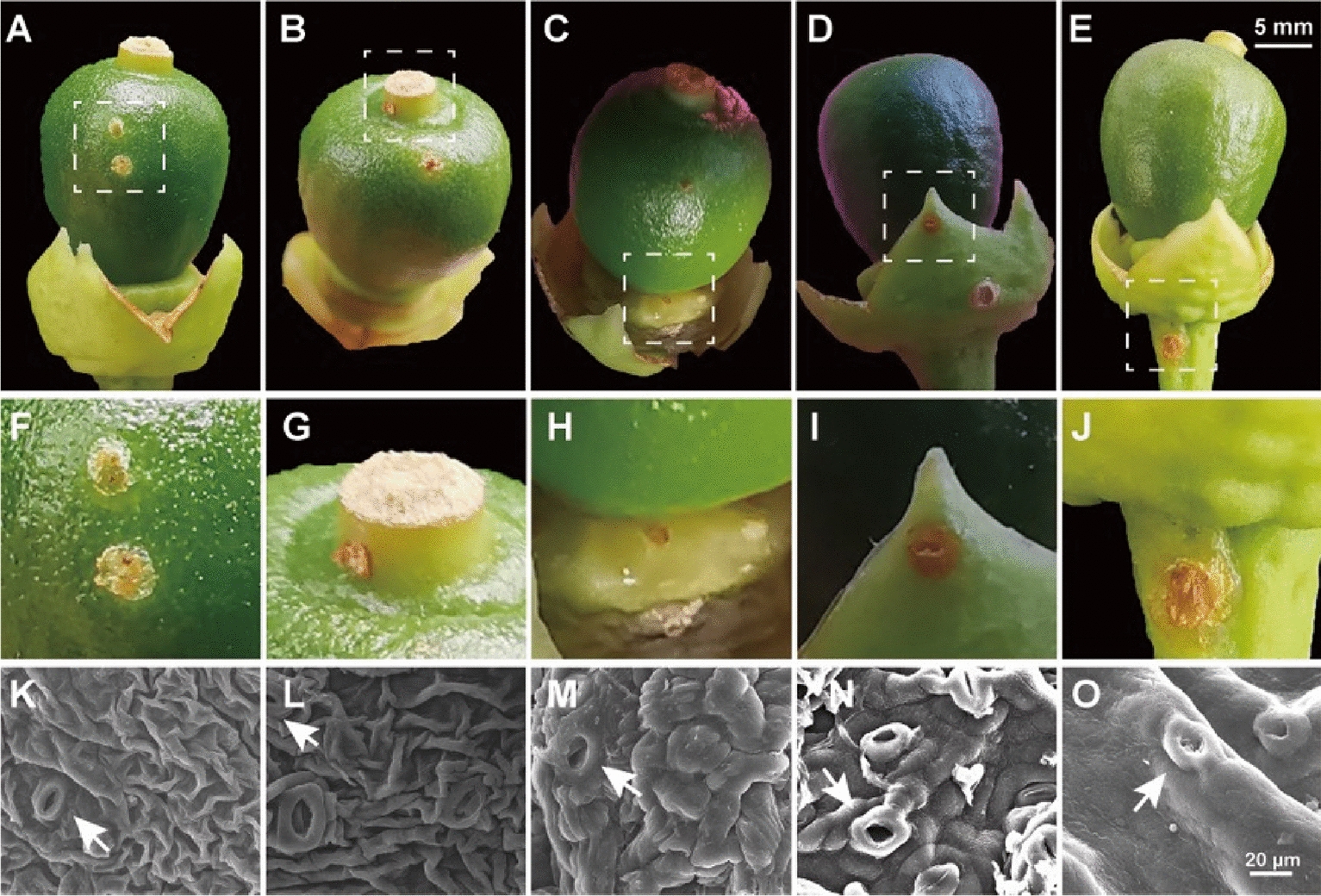
Observation of the canker symptoms on various parts of citrus flower and young fruit. Observed canker symptoms on fruit parts, including **A** epicarp, **B** style residue, **C** nectary disk, **D** sepals and **E** peduncle. **F**–**J**, Enlargements of the same tissue with (**A**–**E**). **K**–**O** SEM images of the same tissue with (**A**–**E**) and the white arrow indicates the stomata

During the blossoming period in a BT sweet orange orchard, young fruits were carefully examined with a magnifying glass to detect the earliest initiation of canker symptoms. Various stages of canker disease symptoms developed 10 to 15 days after petal drop on all the parts of the young fruit, including sepal, style residue, nectary disk, epicarp, and peduncle. Concurrently, developed stomata were detected using SEM in each symptomatic part (Fig. 1).

**Fig. 2:**
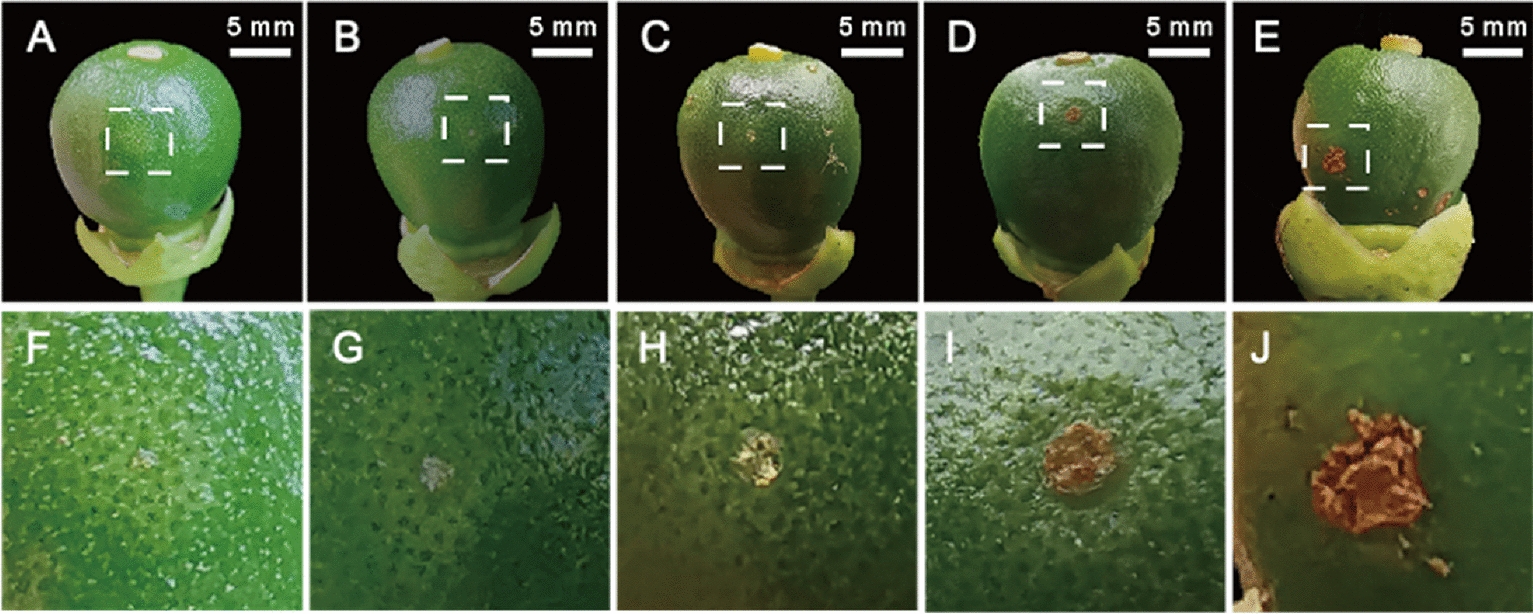
Development of *Xcc* infected symptoms on young fruits. **A**–**E** Observed canker lesions of various degrees on young fruit, ranging from tiny dots to lignification symptoms. **F**–**J**, Enlargements of the dashed white square area in (**A**–**E**)

**Fig. 3:**
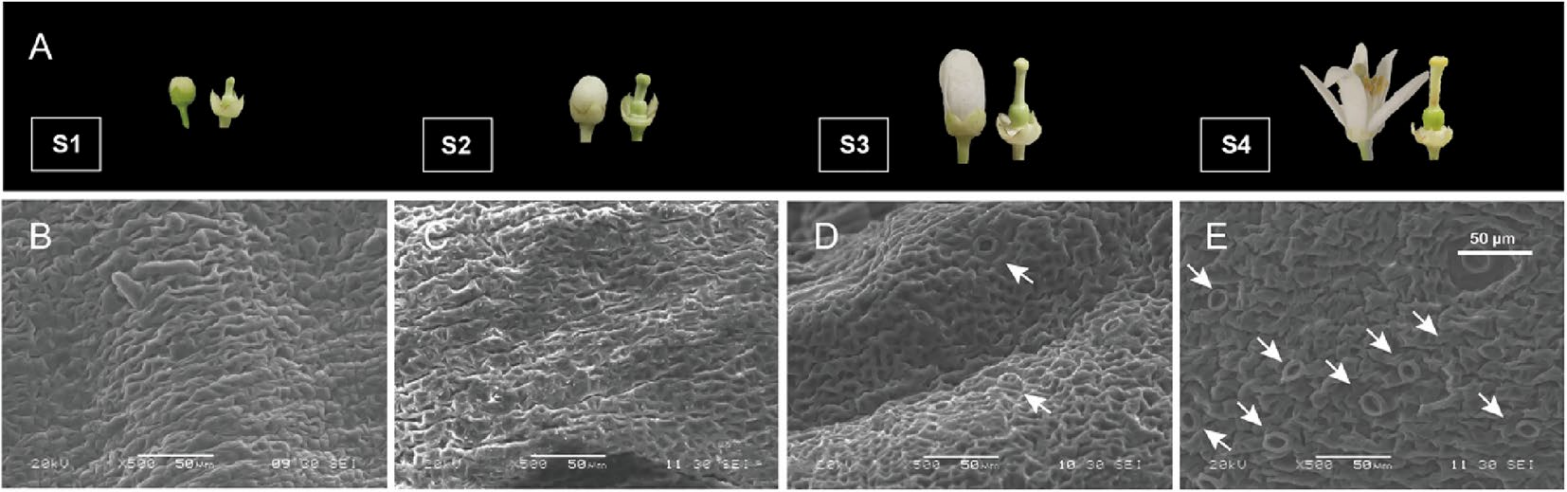
The formation of stomata on the ovary. **A** Flower and ovary corresponding to stomatal observation period, covering (S1) petal appearance, (S2) half petal stage, (S3) full petal stage, and (S4) blossom stage. **B**–**E** Stomata development observed by SEM at S1–S4 flower stages. White arrows indicate stomata

Canker lesions of varying severity, ranging from tiny dots to lignification symptoms, simultaneously appeared on the young fruits at the same developmental stage, indicating earlier infection happened (Fig. 2). Therefore, the formation of stomata on the ovary was further examined, it was revealed that the stomata initiated to form on the ovary at the full petal flower stage (S3) and successively became more abundant. Matured stomata were observed later at the blossom period (S4) (Fig. 3). Thus, it seemed that *Xcc* might have the potential to invade the ovary at the blooming stage of sweet orange.

### *Xcc* invasion during the whole fruit developmental stages but not in the ripening period

**Fig. 4:**
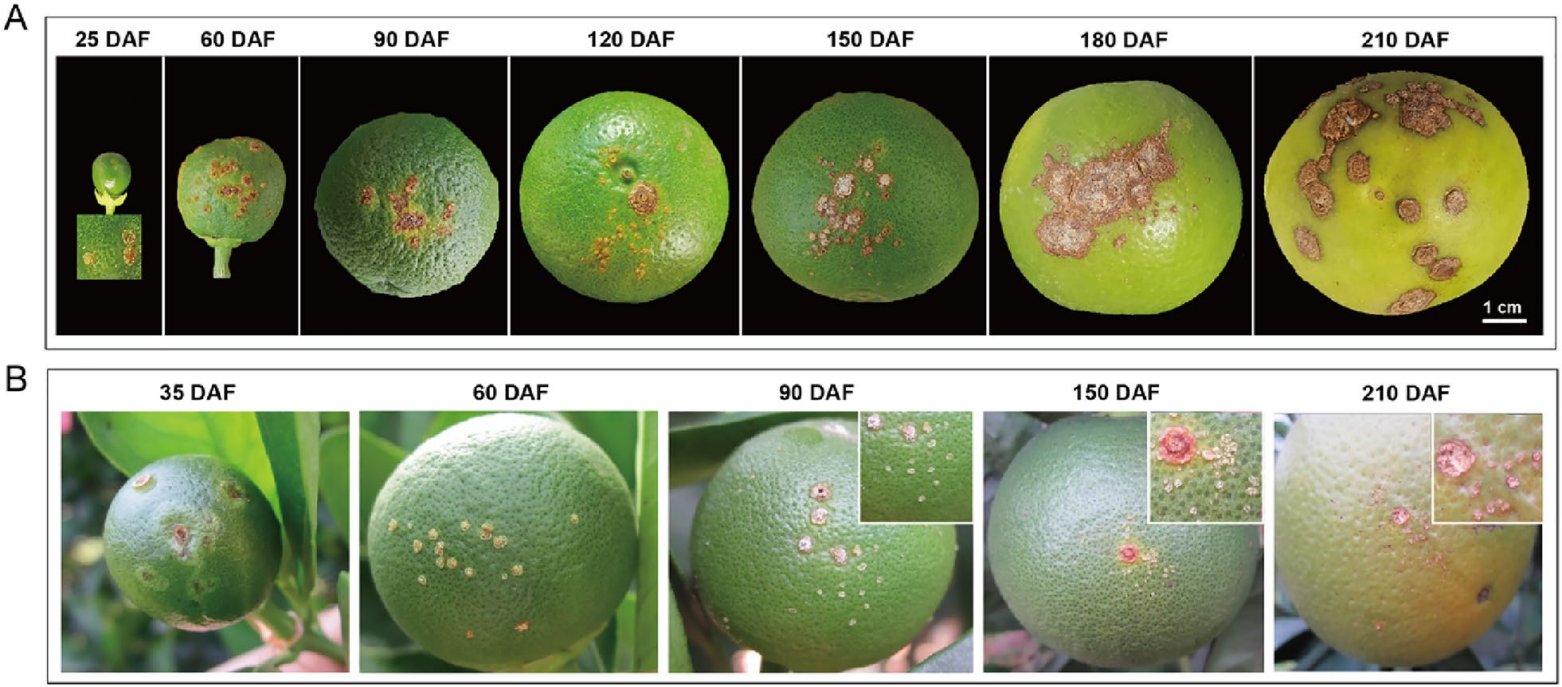
Field investigation of *Xcc* invasion in sweet orange fruits during the full developmental cycle. **A** Canker disease symptoms on fruit during the developmental cycle. **B** Newly formed lesions on fruit during the developmental cycle. The investigation was conducted in the sweet orange production area of Chenzhou City, Hunan Province, China, in the northern hemisphere, starting in May and ending in November. In the figure, 25 DAF to 35 DAF is at the end of local spring, 60 DAF to 120 DAF is in summer, and 180 DAF to 210 DAF is in fall and winter

Sweet orange fruit in the south of Hunan province grows from May to November, and the three stages of fruit entire growth cycle are: the cell division stage in May and June, fruit expansion from July to September, and ripening stage in October and November. In a field survey, *Xcc* infection process was monitored throughout all the fruit developmental stages. At the cell division stage, the lesions predominantly exhibited callus symptoms with slight lignification. During the fruit expansion period, lesions of various developmental degrees were mixed on the pericarp. As the fruit went into maturation, almost no newly formed lesions were observed meaning the infection stopped (Fig. 4).

### In vitro artificial inoculation of *Xcc* on citrus fruit

**Fig. 5:**
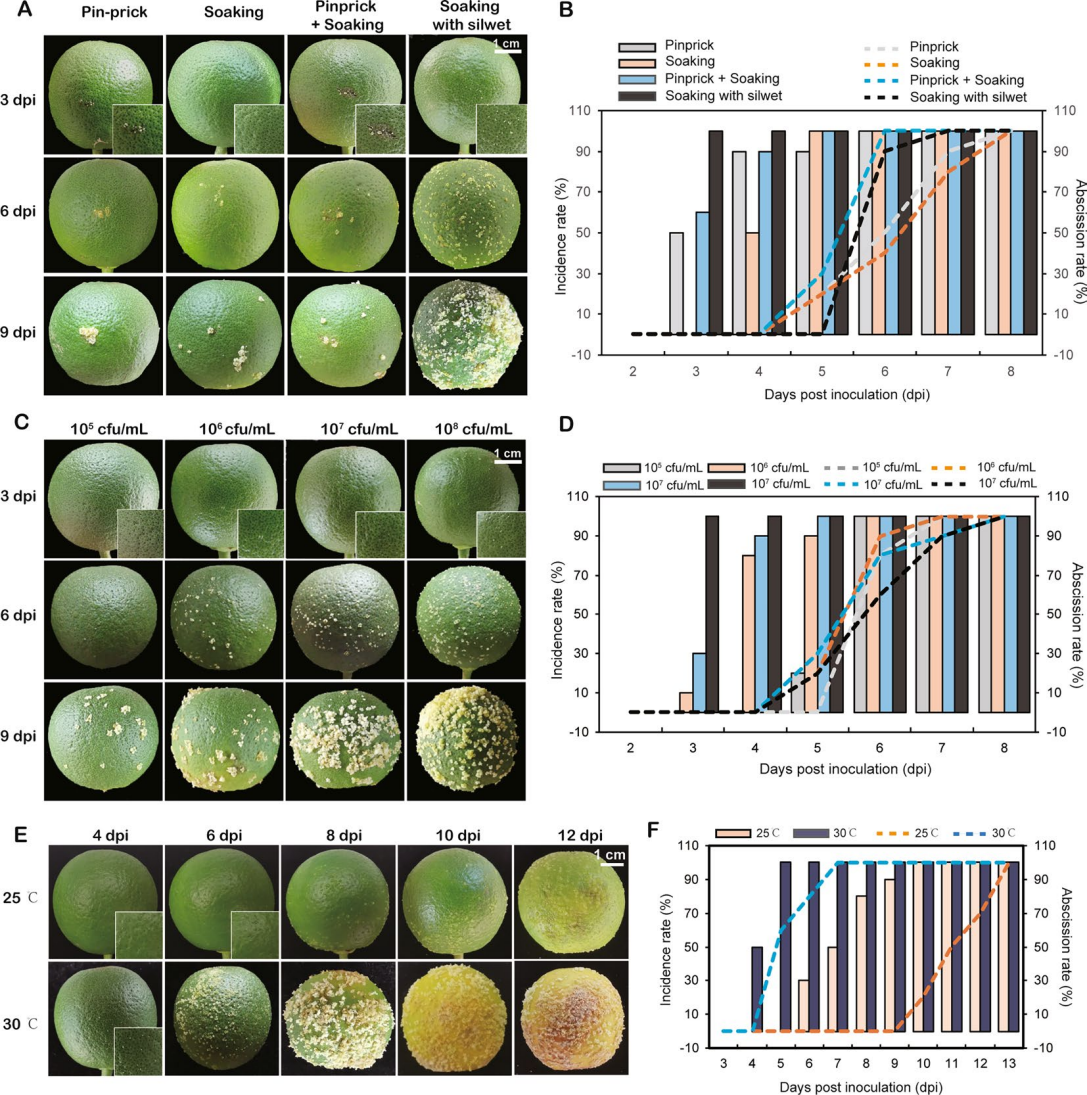
Observation on the canker disease symptoms of the detached fruits by artificial inoculation. **A** Sweet orange fruits inoculated with 10^8^ cfu/mL of *Xcc* by pinprick, soaking, pinprick + soaking and socking with silwet on 3, 6 and 9 dpi. **B** Incidence and abscission rate on sweet orange fruits inoculated with 10^8^ cfu/mL of *Xcc* by pinprick, soaking, pinprick + soaking and socking with silwet. **C** Symptoms of on sweet orange fruits inoculated with 10^5^–10^8^ cfu/mL of *Xcc* by socking with silwet on 3, 6 and 9 dpi. **D** Incidence and abscission rate on sweet orange fruits inoculated with 10^5^–10^8^ cfu/mL of *Xcc* by socking with silwet. **E** Symptoms on sweet orange fruits inoculated with 10^8^ cfu/mL of *Xcc* by socking with silwet incubated at 25 and 30 ℃ on 4, 6, 8, 10 and 12 dpi. **F** Incidence and abscission rate on sweet orange fruits inoculated with 10^8^ cfu/mL of *Xcc* by socking with silwet incubated at 25 and 30 ℃. The bar graph represents the incidence rate, and the dashed line graph represents the abscission rate in (**B, D, F**)

In the in vitro artificial inoculation, detached sweet orange fruits with peduncle were inoculated with 10^8^ cfu/mL of *Xcc* using four methods, i.e. pinprick, soaking, soaking + pinprick, and soaking with silwet. The peduncle was required to keep fruit fresh during in vitro inoculation. Consequently, the abscission rate between fruit and peduncle was calculated for all four methods. The disease symptoms on fruit were periodically observed, and it was found that the lesion area and callus accumulation gradually increased over time. Specifically, on the fruits inoculated by soaking with silwet, symptom uniformly initiated on 3 dpi and were the earliest to reach 100% infection rate. Although fruits inoculated by pinprick and soaking had slower abscission rate, both also exhibited lower onset uniformity (Fig. 5A, B).

*Xcc* inoculum concentrations were tested for speed of symptoms appearance and number of disease spots on citrus fruit. Fruits inoculated with 10^8^ cfu/mL of *Xcc* all showed symptoms on 3 dpi, with fine callus lesions observed on the fruit surface, but those inoculated with 10^5^ cfu/mL did not show mild symptoms until 5 dpi. In terms of fruit abscission, the process was relatively similar among the four inoculum concentrations, and all the treated fruits completely dropped on 8 dpi (Fig. 5C, D).

Temperature is a key factor affecting not only the occurrence of canker disease but also the inoculation cycle. The inoculated fruits incubated at 30 ℃ showed typical symptoms first on 4 dpi (Fig. 5E). While those under 25 ℃, symptoms occurred 5 days later. Soon afterwards, damage appeared on fruit surface and the fruit completely fell off (Fig. 5E, F). Although fruits could be successfully inoculated with *Xcc* under 25 ℃, the disease development was slow and typical symptoms did not manifest.

A comprehensive approach combining treatment method, inoculum concentration and incubation temperature generally resulted in high-efficiency inoculation by soaking with 10^8^ cfu/mL bacterial suspension containing silwet at 30 ℃, facilitating short-term in vitro research on citrus fruit canker disease.

### In vivo artificial inoculation of *Xcc* on citrus fruit

**Fig. 6:**
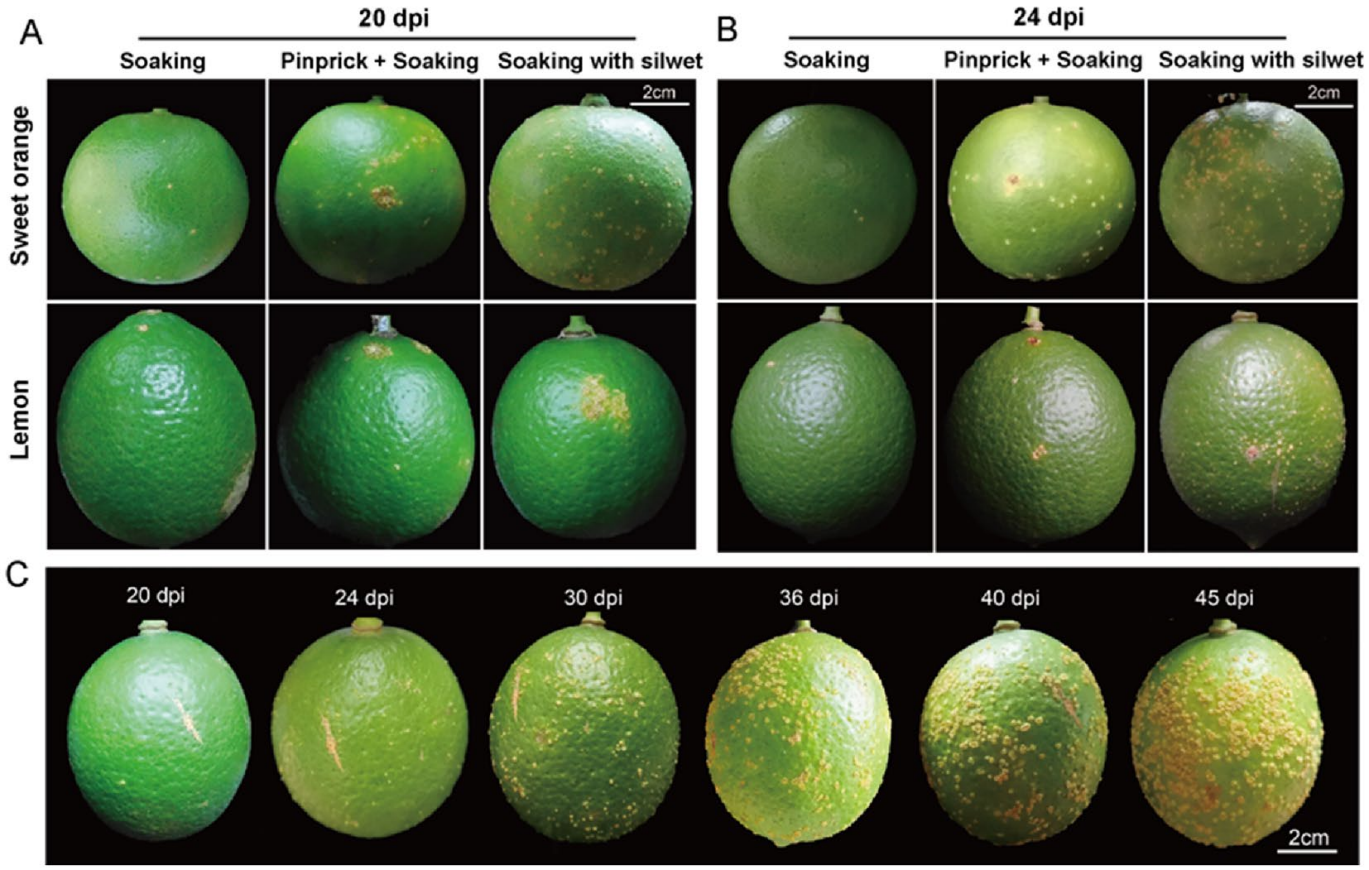
Symptoms of sweet orange and lemon fruits inoculated with *Xcc* by in vivo inoculation. **A** and **B** Fruits were sampled at about 120 DAF and inoculated with 10^8^ cfu/mL of *Xcc* by soaking, pinprick + soaking and socking with silwet. **C** The inoculated lemon fruits lasted for 45 days

Given that the short cycle of in vitro inoculation is insufficient for prolonged symptom observation because of the increased difficulty of *Xcc* invasion in the later stages of fruit development, in vivo inoculation was conducted on two susceptible cultivars, BT sweet orange and ‘Eureka’ lemon, and young fruits at 120 DAF were treated. Inoculation by soaking, soaking + pinprick, and soaking with silwet gave successful results for both cultivars. Similar to in vitro inoculation, soaking with silwet demonstrated the best results among the three methods. However, obvious differences were observed between two genotypes. The inoculated sweet orange fruits showed typical lesions on 20 dpi, while on those of did not appear till 24 dpi. On the other hand, sweet orange fruits were more likely to abscise than lemon fruits, which remained over 45 days before abscise occurred. (Fig. 6).

### Canker symptom observation during all fruit developmental stages following artificial inoculation

**Fig. 7:**
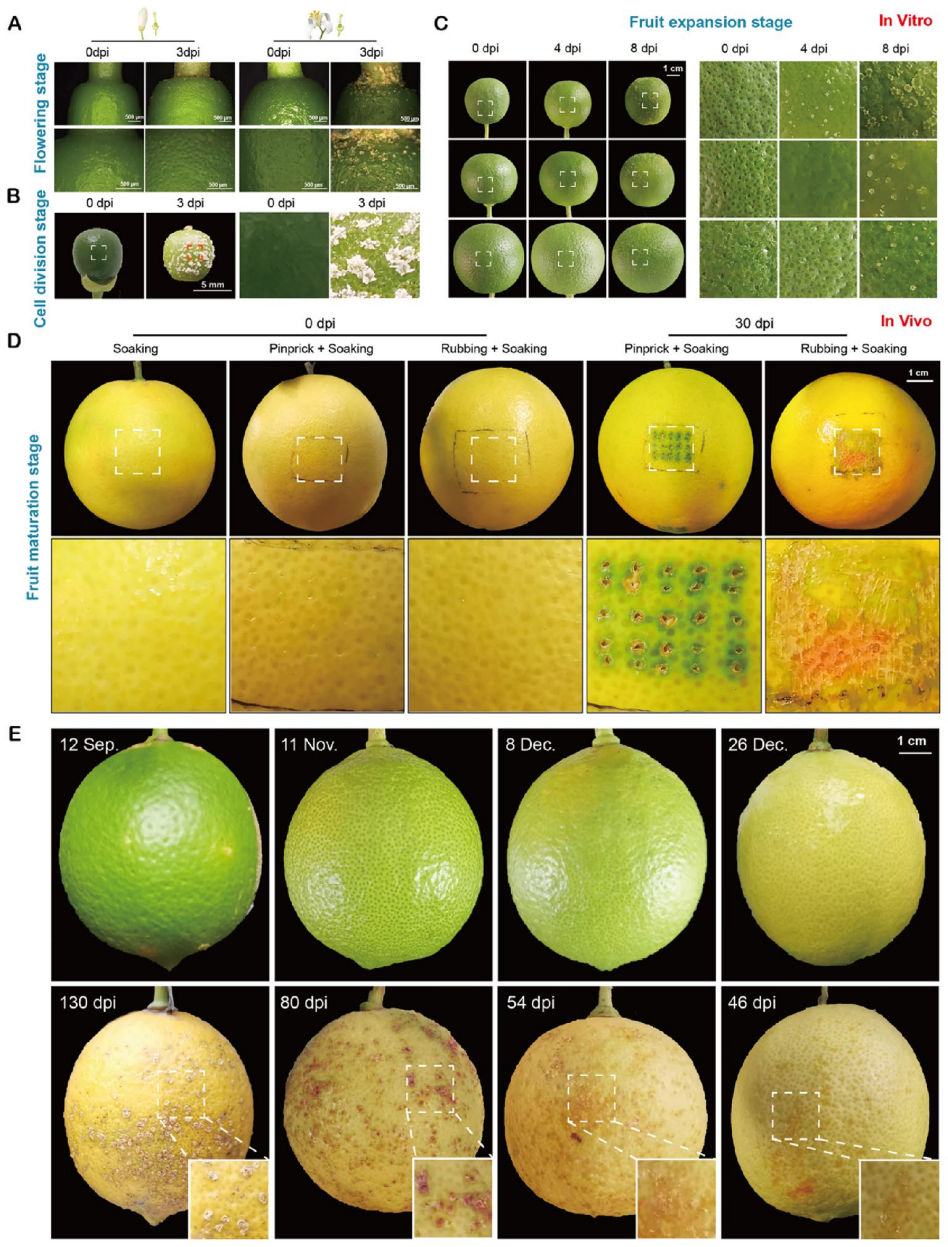
Observation on the canker disease symptoms in the whole fruit development stage by artificial inoculation. **A** Symptoms on sweet orange ovaries in vitro inoculated with *Xcc* at flowering stage. **B** Symptoms on sweet orange fruits in vitro inoculated with *Xcc* at cell division stage. **C** Symptoms on sweet orange fruits in vitro inoculated with *Xcc* at expansion stage. **D** Symptoms of *Xcc* inoculated sweet orange fruits at maturation stage in vivo. Fruit surfaces other than pinprick or rubbing were also inoculated with *Xcc* by soaking with silwet. **E** Symptoms on lemon fruits in vivo inoculated with *Xcc* at maturation. All the inoculation was performed by soaking fruits in 10^8^ cfu/mL of *Xcc* containing silwet

To confirm the regularity of canker disease of fruits observed in field, artificial inoculation of *Xcc* was performed under controlled conditions throughout the developmental cycle of sweet orange fruit. Inoculation began at the full petal flower stage. On the 3 dpi, the calli-like symptoms were observed on the ovary wall. More and more pronounced symptoms appeared at the flowering stage (Fig. 7A). Fruits seemed more sensitive to *Xcc* during cell division (Fig. 7B), and infection became progressively more difficult as the fruit into maturation (Fig. 7C). At the ripening stage, the canker symptoms on fruit surface were not observed on the 30th day after in vivo inoculation, even when the surface structure the was physically disrupted by needle dip and friction dip (Fig. 7D). However, lemon fruits turned yellow in color were still infected (Fig. 7E), indicating different responses in two cultivars to *Xcc*.

### Monitoring *Xcc* invasion process in fruit

**Fig. 8:**
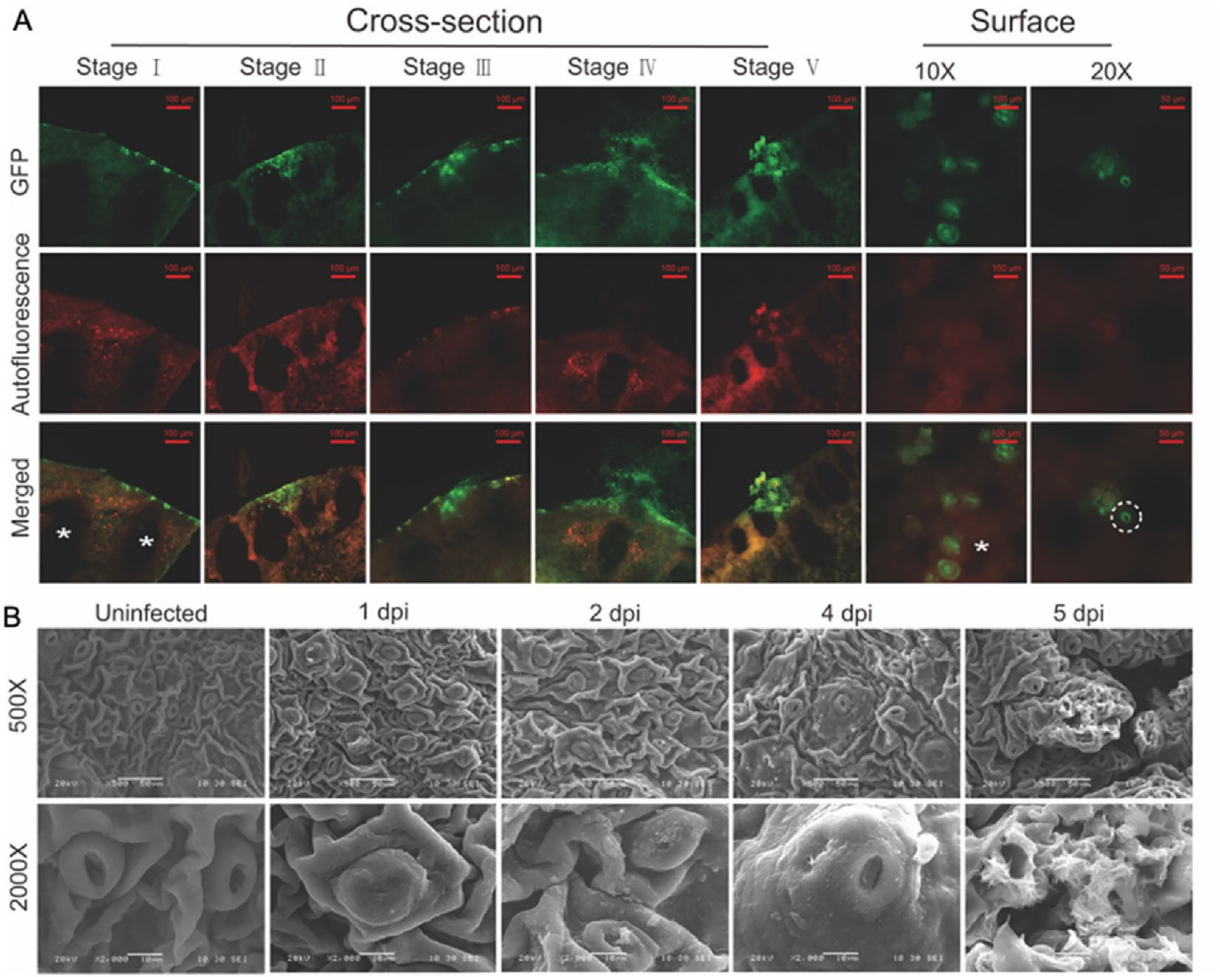
Monitoring the process of eGFP*-Xcc* invasion on sweet orange fruits. **A** The cross-section of fruits at 30 DAF on 3 (Stage I, II, III) or 6 dpi (Stage IV, V) and fruit surface on 6dpi inoculated with eGFP*-Xcc* observed by fluorescence microscope. **B** The surface of fruits 80 DAF inoculated with *Xcc* invasion observed by SEM. All fruits were inoculated with 10^8^ cfu/mL of *Xcc* by soaking with silwet. White * represented oil glands and white dashed circle represented stomata

Process of the pathogen invasion into fruits was monitored by inoculation with eGFP-*Xcc*. As seen in the Fig. 8A, the bacteria first attached to the epicarp surface, gathered at the invasion site (Stage I), gradually invaded inward (Stage II), and then penetrated the epidermis (Stage III, IV) to form calli-like lesions (Stage V). The lesions were also visible on the epicarp surface with pronounced fluorescence, suggesting significant *Xcc* enrichment. Additionally, fluorescence was observed around the stomata (Fig. 8A), indicating that *Xcc* invaded through this entry point.

The modification of fruit surface structure caused by pathogen invasion was observed by SEM. Compared with uninoculated fruit, the infected surface produced irregular crystalline materials, and the stomata were partially closed or blocked by secretions. As the surface materials increased, a bulge was formed outward from the stomata successively. Then, it broke through the epidermis, causing fruit surface cracking and forming disease lesions (Fig. 8B).

### Evaluation of tolerance of citrus genotypes to *Xcc* infection by in vitro artificial inoculation on fruit

**Fig. 9:**
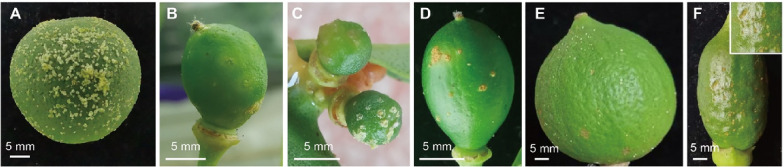
Symptom comparison among citrus genotypes by in vitro artificial inoculation on fruit. **A** Symptoms of ‘Bingtang’ sweet orange on 6 dpi. **B** Symptoms ‘Huapi’ kumquat on 6 dpi. **C** Symptoms of Hongkong kumquat on 8 dpi. **D** Symptoms of ‘Rong’an’ kumquat on 24 dpi. **E** Symptoms of round citron on 18 dpi. **F** Symptoms of ‘C-05’ citron on 20 dpi. All fruit inoculated with 10^8^ cfu/mL of *Xcc* by soaking with silwet

The reported resistant genotypes, like Hongkong kumquat (*Fortunella hindsii*), ‘Rong’an’ and ‘Huapi’ kumquat (*F. crassifolia*), citron C-05 and Round citron (*C. medica*), were collected at the young fruit stage and evaluated by soaking in 10^8^ cfu/mL of *Xcc* suspension containing silwet and incubated at 30 ± 1 ℃, in comparison with the fruits of BT sweet orange. Young fruits of all tested genotypes were successfully inoculated and differences in resistance were clearly identified. The susceptible BT sweet orange manifested disease spots covering the whole fruit on 6 dpi. At the same time, Hongkong kumquat fruits presented many callus lesions, while on ‘Huapi’ and ‘Rong’an’ kumquat fruits only presented a few lesions. ‘Rong’an’ kumquat fruits were observed for more than 24 dpi and the lesions developed into typical canker symptoms. Citron C-05 and Round citron did not show any typical symptoms, similar to the reaction previously reported on leaf tests [26]. Although few callus-like lesions appeared in both citrons at the beginning of inoculation, but no further symptom development continued (Fig. 9).

## Discussion

Citrus canker disease, when it infects fruits, significantly damages the benefits of the citrus industry. Unfortunately, there is no commercially cultivated citrus variety worldwide possessing undisputed immunity to *Xcc*. Precise prevention and control strategies remain the most efficacious and beneficial measures for controlling canker disease. The protracted growth cycle and intricate structure of citrus fruits have imposed limitations on canker research. Consequently, there exists a critical imperative to systematically investigate the intricate interplay between citrus fruit development and the infection patterns orchestrated by *Xcc*. Presently, the researchers have concentrated on *Xcc*-leaves interaction, leading to a considerable knowledge gap concerning the infection dynamics in fruits and the corresponding response of *Xcc*. Furthermore, the absence of a well-established method system for fruit inoculation further impedes the research progress on citrus fruit reaction to canker disease.

In horticultural plants, the research on fruit diseases mainly focuses on postharvest fungal diseases, such as blue and green molds of citrus [27], ring rot disease of apple [28], and grey mold of tomato [29] etc. These pathogenic fungi are usually inoculated by injecting suspended spore solution or inserting agar disk containing mycelium. However, citrus canker is s a bacterial disease and the reported inoculation methods, injection, pinprick, and spray techniques, are limited from leaf inoculation [30, 31].

To establish an efficient *Xcc* inoculation system on fruits, a series of trials were conducted by both in vivo and vitro inoculations using different methods. Unlike on leaf, citrus fruit exhibits external and internal divisions into epicarp, albedo, segment membrane, and juice sacs layers. *Xcc* primarily targets the epicarp of citrus fruits, but the inherent high fruit surface osmotic pressure resulting from the oil gland forms obstacles for injection-based inoculation. Therefore, pinprick, soaking, pinprick + soaking, and soaking with silwet were tested to facilitate pathogen invasion of the outer tissues. The results indicated that single pinprick or soaking inoculation could delay fruit detachment from the fruit stalk, but pinprick inoculation had limitations as it was suitable only for localized inoculation and required careful manipulation to control the pinprick intensity for different citrus varieties with varying peel thicknesses. While soaking alone proved to be limited in attaching bacteria to the fruit surface due to water surface tension, the addition of surfactants addressed this limitation. The combination of pinprick and soaking failed to overcome the issue of low effective inoculation due to water surface tension, but exacerbated fruit detachment. Among the tested methods, soaking with silwet emerged as the most efficient, displaying uniform disease development and coverage (Fig. 5). Silwet L-77, a silicone surfactant known for substantially reducing water surface tension, effectively wets the surface of various plant types, significantly enhancing the coverage of target organisms [32, 33]. Notably resistant to water scouring and penetration, Silwet L-77 has been widely employed as a surfactant in transgenic studies involving *Arabidopsis thaliana* [34, 35] and other crops [36]. In this study, the inclusion of Silwet L-77 as a surfactant played a crucial role in facilitating the attachment and penetration of *Xcc* on the surface of spherical fruits, resulting in efficient infection. In vivo inoculation also confirmed the effectiveness of this method (Fig. 6). However, during the inoculation process, we found that as the fruits develop, the difficulty of inoculation increased, and in vitro inoculation cycle could not meet the requirements for observing symptoms in the late stage of fruit development. Hence, we suggested evaluating young fruit using in vitro inoculation, while in vivo inoculation became more suitable for observing citrus canker disease symptoms as the fruit reached to the expansion and ripening stages.

The complete development cycle of citrus fruit initiates with bud emergence and culminates in harvesting. Field observations and artificial inoculations conducted in this study clarified that *Xcc* could initiate infection on sweet orange fruits as early as at the flowering stage, with the increasing invasion during fruit expansion stage. The infection became challenging as the fruit develops, and ultimately being stopped at the ripening stage (Fig. 7). This pattern is consistent with previous reports, indicating that the resistance to citrus canker increases with the age of the tissue [37–39].

Graham [5] reported that high infection rates of *Xanthomonas campestris* pv. *citri* (*Xcc*) strains were observed in sweet orange fruits with diameters between 20 and 40 mm, while infection rates became lower for fruits below or beyond this range. Further report indicated that very young tissues resist *Xcc* invasion due to their immature stomata, and fruits during the later growth expansion stage are most susceptible to infection [4]. The present study revealed more specific key time points for invasion. Contrast to previous reports, typical lignified canker lesions appeared on young fruits approximately 10 days after blossom dropping, indicating the invasion had already commenced. The early lesion development involved continuous enlargement and lignification (Fig. 2). Even under favorable conditions, a minimum of 7 days were required for tiny spots to appear, extending to 60 days under unfavorable conditions. The present results suggested that *Xcc* invaded the ovary at the time of full bloom in sweet orange, leading to the appearance of lignified spots on young fruits after flowering. Successive artificial inoculation experiments (Fig. 7A) confirmed the invasion of *Xcc* to citrus fruit 2–3 weeks earlier than previous reported observation [4]. This phenomenon might relate with global warming, as the pathogen may find an optimum temperature for infections during spring, a season previously considered unsuitable for *Xcc* infection [19]. Based on these results, it essential to start the prevention and control of citrus canker disease from the flowering stage and extend until fruit ripening.

Furthermore, it is generally believed that the accumulation of plant surface substances, such as waxes, is perceived as a natural barrier against successful invasion. Leaves, twigs, and fruits develop thickened cuticles during maturation to increase resistance to *Xcc* invasion. However, our study revealed that *Xcc* failed to infect mature sweet orange fruits despite breaking down the surface physical barriers. In contrast, mature lemon fruits were easily infected under suitable condition (Fig. 7E). It means that, in addition to physical factors, there may be other mechanisms linked to the pathogen invasion. Koizumi inoculated *Xanthomonas campestris* pv*. citri* onto wounded leaves to study the relationship between wound-healing process of citrus leaf tissues and successful infection by observing tissue structures. The results indicated that the formation of a thin dividing cell layer and lignified cells after wound inhibited pathogen invasion, but this effect was influenced by humidity and temperature [40]. Additionally, using electron microscopy to observe the ultrastructure of infected leaf tissue cells, the researcher also found that in resistant varieties, bacteria could be coagulated and encapsulated by fibrous materials after invasion to restrict their spread [41]. Thus, the interactions between citrus plants and *Xcc* are quite complex, and more intricate related to fruit infection.

Pathogens enter intercellular space of the plant tissue through open channels such as stomata and wounds [42]. In this study, we observed that fruit (ovary) initiates the formation of stomata during the full petal flower stage (Fig. 3), and subsequent artificial inoculation confirmed that *Xcc* infection to the ovary during this stage. Intriguing, stomata were present in various parts of young fruits, including the epicarp, residual style, sepals, and flower disc. Concurrently, the appearance of spots was observed on these tissues for the first time in field orchards (Fig. 1). Observation of eGFP*-Xcc* by fluorescence microscopic in this study unveiled bacteria clustering around stomata, suggesting a potential invasion pathway (Fig. 8). These results demonstrate that the formation of stomata in undamaged tissues provides a critical entry point for *Xcc* to invade the tissues internally. On leaves, similar to bacterial diseases in most plants, *Xcc* typically invades through the lower epidermis, where stomatal density is higher. The examination of stomatal structure and density across different cultivars revealed independence from cultivar resistance, yet the number of bacteria traversing stomata aligned with the final spot count, suggesting that individual lesions originated from stomatal infections [5], and a significant accumulation of *Xcc* in leaf stomata 5 days later after spraying [2]. Our previous studies found that *Xcc* aggregated around the lower epidermis stomata of sweet orange leaves, confirming stomata as one of the channels for *Xcc* to enter the intercellular spaces. Additionally, spray inoculation tests on leaves of different ages indicated that *Xcc* invasion started from young leaves, with the leaves of at half their maximum area exhibiting the highest sensitive. As leaves mature, they become less susceptible. This result was correlated with stomatal development, which were under development in young tissues and became smaller in the gap in the stomata of the old mature tissues [16]. Our research on fruit stomata mirrors leaf studies, emphasizing the importance of stomatal development in *Xcc* invasion [15].

Over an extended period, the selection and breeding of varieties resistant to canker disease have been carried out [43]. Currently, in vivo leaf inoculation served as the primary approach for assessing the susceptibility or resistance of citrus germplasms to citrus canker disease, offering a more effective reflection of the plant's sensitivity to *Xcc* [44]. Following a decade of intensive exploration, our research group has identified a genotype in citrus, citron C-05, highly resistant to *Xcc* [45]. In this study, we artificially inoculated fruits of various genotypes with a high concentration of bacteria solution. Relative to sweet oranges, kumquat fruits (*F. crassifolia*) exhibited a degree of resistance consistent with leaf findings [46, 47]. However, HongKong kumquat fruits displayed distinct and high-density canker disease lesions. This outcome might be attributed to the uneven surface of tender and small kumquat fruit, which enrich a higher inoculum concentration, thereby overcoming their limited resistance [48]. On the other hand, Round citron and citron C-05 fruits manifested restricted lesion expansion maintaining the same level of resistance as observed in leaves. Notably, citron C-05 also presented sunken necrotic lesions similar to leaves [26]. Based on these results, It may be conclude that under conditions ensuring a relatively consistent fruit development cycle among germplasms, fruit inoculation effectively distinguishes resistant and susceptible varieties. When the stability of disease resistance evaluations is in question, lower inoculation concentrations may be utilized to enhance sensitivity. However, it is important to note that the citrus germplasms evaluated in this study were limited, and the substantial differences in physical structures, stomatal density, and other conditions between leaves and fruits of different germplasms may not fully reflect the responses of all citrus germplasms to *Xcc* after inoculation. Nevertheless, the combined evaluation of leaves and fruits provides a more comprehensive understanding of the response of different germplasms to *Xcc*.

## Conclusion

After *Xcc* invades the early fruit during the flowering stage in the field, and the infection persists throughout the fruit development cycle until fruit ripening. The clarification of this pattern will be helpful to guide prevention and control to citrus canker disease of sweet orange. The efficient in vivo and in vitro inoculation system for fruit with *Xcc* was established, and it is useful for disease resistance/susceptibility evaluations. Also, the artificial inoculation of *Xcc* on developmental cycle of sweet orange confirmed the invasion pattern observed in field. The results also indicated that the resistance to *Xcc* was not caused by environmental factors and fruit surface barriers. In the future, we will focus on the mechanism of sweet orange ripening fruit resistance to canker disease in order to excavate canker resistance genes and characterize inhibitory secondary metabolites for control in production.

## Data Availability

Not applicable.
